# Thioredoxin h2 inhibits the MPKK5-MPK3 cascade to regulate the CBF–COR signaling pathway in *Citrullus lanatus* suffering chilling stress

**DOI:** 10.1093/hr/uhac256

**Published:** 2022-11-21

**Authors:** Anqi Xu, Nannan Wei, Hao Hu, Shu Zhou, Yuan Huang, Qiusheng Kong, Zhilong Bie, Wen-Feng Nie, Fei Cheng

**Affiliations:** Key Laboratory of Horticultural Plant Biology, Ministry of Education, College of Horticulture and Forestry Sciences, Huazhong Agricultural University, Wuhan 430070, China; Key Laboratory of Horticultural Plant Biology, Ministry of Education, College of Horticulture and Forestry Sciences, Huazhong Agricultural University, Wuhan 430070, China; Key Laboratory of Horticultural Plant Biology, Ministry of Education, College of Horticulture and Forestry Sciences, Huazhong Agricultural University, Wuhan 430070, China; Key Laboratory of Horticultural Plant Biology, Ministry of Education, College of Horticulture and Forestry Sciences, Huazhong Agricultural University, Wuhan 430070, China; Key Laboratory of Horticultural Plant Biology, Ministry of Education, College of Horticulture and Forestry Sciences, Huazhong Agricultural University, Wuhan 430070, China; Key Laboratory of Horticultural Plant Biology, Ministry of Education, College of Horticulture and Forestry Sciences, Huazhong Agricultural University, Wuhan 430070, China; Key Laboratory of Horticultural Plant Biology, Ministry of Education, College of Horticulture and Forestry Sciences, Huazhong Agricultural University, Wuhan 430070, China; Department of Horticulture, College of Horticulture and Plant Protection, Yangzhou University, Yangzhou 225009, China; Key Laboratory of Horticultural Plant Biology, Ministry of Education, College of Horticulture and Forestry Sciences, Huazhong Agricultural University, Wuhan 430070, China

## Abstract

Thioredoxins (TRXs) are ubiquitous oxidoreductases and present as a multigenic family. TRXs determine the thiol redox balance, which is crucial for plants in the response to cold stress. However, limited knowledge is available about the role of TRXs in watermelon (*Citrullus lanatus*), which is highly sensitive to chilling stress in agricultural practice. Here, we identified 18 genes encoding 14 typical and 4 atypical TRXs from the watermelon genome, and found that ClTRX h2 localized at the plasma membrane was largely induced by chilling. Virus-induced gene silencing of *ClTRX h2* resulted in watermelon plants that were more sensitive to chilling stress. We further found that ClTRX h2 physically interacted with mitogen-activated protein kinase kinase 5 (ClMPKK5), which was confirmed to phosphorylate and activate ClMPK3 *in vitro*, and the activation of ClMPK3 by ClMPKK5 was blocked by a point mutation of the Cys-229 residue to Ser in ClMPKK5. Additionally, ClTRX h2 inhibited the chilling-induced activation of ClMPK3, suggesting that the ClMPKK5–ClMPK3 cascade is regulated in a redox-dependent manner. We showed that *ClMPK3-*silenced plants had increased tolerance to chilling, as well as enhanced transcript abundances of the *C-repeat/DREB binding factor* (*ClCBF*) and *cold-responsive* (*ClCOR*) genes. Taken together, our results indicate that redox status mediated by ClTRX h2 inhibits ClMPK3 phosphorylation through the interaction between ClTRX h2 and ClMPKK5, which subsequently regulates the CBF–COR signaling pathway when submitted to chilling stress. Hence, our results provide a link between thiol redox balance and MAPK cascade signaling, revealing a conceptual framework to understand how TRX regulates chilling stress tolerance in watermelon.

## Introduction

Serious cell damage caused by chilling stress frequently occurs when plants are exposed to surrounding temperatures that are much lower than the optimal growth temperature. Cellular oxidation leads to cell damage, which not only influences plant growth but also limits stress responses. Oxidative signals or oxidation–reduction cycles are necessary for activating an adaptive response to sense the varied environmental conditions [1]. In plants, thiol-containing proteins are essential for integrating signals generated by reductive metabolism and reactive oxygen species (ROS) network-mediated electron drainage [2]. Additionally, thiol oxidation results in multiple functional levels of regulation, including metabolic enzymes, signal transduction elements, and transcription and translation factors [3].

Thioredoxin (Trx/TRX) is a kind of small ubiquitous protein, containing a conserved WC(G/P)PC motif enabling a dithiol–disulfide exchange reaction with targeted proteins [4]. Specifically, Trx interacts with multiple proteins to regulate their structures and function in a dynamic manner and thus participates in different signaling pathways. For example, Trx was reported to negatively regulate the apoptosis signal-regulating kinase 1 (ASK1/MAPKKK5) through interacting with the non-catalytic N-terminal and forming a Trx–ASK1 complex without phosphorylation activity; oxidation at the positions of Cys32 and Cys35 of Trx1 forms a disulfide bond that dissociates ASK1, leading to activation of ASK1 and the downstream cell apoptosis pathways in humans [5, 6]. In addition, paraquat-induced oxidative stress causes Trx oxidation, which activates ASK1 and the following JNK and p38 MAPK (mitogen-activated protein kinase) pathways to induce apoptosis [7]. Recently, a cell-permeable fusion protein, Tat–Trx1, was reported to reduce inflammation by inhibiting lipopolysaccharide-induced activation of MAPK and NF-κB signaling [8].

The *Arabidopsis* TRXs are classified into two main classes based on their active site: the atypical XCXX (C/S) active site and typical WC(G/P)PC active site [9]. Moreover, TRXs could be divided into a number of subclasses according to the primary structure and subcellular localization. The subclasses of typical TRX f, m, x, y, z, and several kinds of atypical TRXs (CDSP32, NADPH thioredoxin reductase C, and ferredoxin-thioredoxin reductase) localize into plastids [10–12]. The typical o-type TRX proteins locate in mitochondria [13], whereas the h-type proteins are primarily cytosolic or mitochondrial [14–16]. Plant TRXs are involved in the oxidative stress response, and an increasing number of studies have shown a role for h-type TRX-mediated redox regulation in cold stress response in *Arabidopsis* and rice. AtTRX h3/AtTRX h5 contributes to the determination of cellular redox status, which is required for the monomerization and subsequent nuclear translocation of NPR1 to attenuate cold-induced oxidative stress in *Arabidopsis* [17]. Furthermore, AtTRX h2-mediated redox variation causes the similar oligomer-to-monomer transition and functional activation of C-repeat/DREB binding factors (CBFs) to enhance cold tolerance [18]. A cold-induced *OsTRX23*/*OsTRX h1* represses the kinase activities of OsMPK3 and OsMPK6 *in vitro* in a redox-dependent manner in rice [19], suggesting a potential link between TRX h and MAPK in response to cold stimulation in the plant. However, the detailed molecular mechanism and biological significance corresponding to TRX h–MAPK interaction in plants is less characterized so far.

As a post-translational modification event, protein phosphorylation frequently occurs when plants are submitted to biotic or abiotic stresses. Recent studies show that protein phosphorylation is important in CBF-dependent signaling in plants [20, 21]. For instance, MPK3/MPK6 phosphorylate the conserved Ser/Thr residues of ICE1 (inducer of *CBF* expression 1), which destabilizes ICE1 and inhibits its transcriptional activation function, thus repressing *CBF* and *cold-responsive* (*COR*) gene expression and increasing freezing susceptibility in *Arabidopsis* [22, 23]. Additionally, another MEKK1–MKK2–MPK4 cascade was reported to promote the expression of *CBF*s and increase tolerance to freezing by inhibiting the MKK4/5–MPK3/6 cascade [23], suggesting that signaling pathways in the regulation of the phosphorylation of MAPK are diverse. However, it is unclear whether redox status mediated by TRX h is essential in regulating MAPK activity in horticultural plants. Furthermore, the connection between TRX h–MAPK and the CBF–COR module in cold signaling has not been well elucidated in plants.

Watermelon (*Citrullus lanatus*) is widely grown in the world. Since watermelon plants are extremely sensitive to low temperatures, chilling stress frequently results in yield loss in agricultural practice. In this study, we firstly identified a plasma membrane-localized ClTRX h2 protein whose gene expression was significantly induced by cold stress, and then showed that ClTRX h2 is involved in the chilling stress response through its interaction with the MAPK signaling cascades, thereby positively regulating chilling tolerance by inhibiting ClMPK3 activity and subsequently inducing expression of *ClCBF*s in watermelon plants.

## Results

### Characterization of the *ClTRX* family genes in *C. lanatus* genome

HMM profile and Pfam analyses identified 18 unique ClTRX-encoding sequences in the Watermelon (97103) v1 Genome Database (Supplementary Data Table S2). The nomenclature of the identified ClTRXs was assigned in accordance with the *Arabidopsis* TRX nomenclature system (Supplementary Data Table S2). The *ClTRX* genes were located on 9 of the 11 chromosomes in watermelon (Supplementary Data Table S2). The CDSs of the *ClTRX* members ranged from 315 bp (*ClTRX z*) to 594 bp (*ClTRX o*), and the corresponding number of amino acids (aa) varied from 104 to 197. Their predicted subcellular localizations were different (Supplementary Data Table S2), suggesting their functional diversity in watermelon. The pI values for the ClTRX proteins varied from 4.22 to 9.51, and the Mw for these was in the range of 12.00 and 21.85 kDa (Supplementary Data Table S2). The active site (redox center) of each ClTRX isoform is also represented in Supplementary Data Table S2.

**Figure 1:**
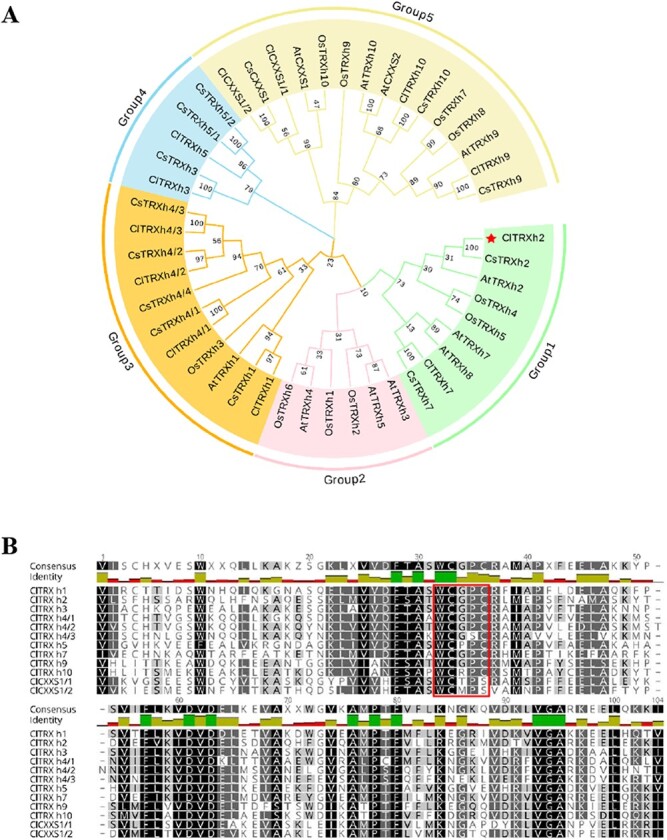
Phylogenetic analysis of h-type TRXs and sequence alignment of 12 h-type ClTRXs in watermelon. (A) Phylogenetic tree representation of h-type TRXs from *Arabidopsis* (At), rice (Os), cucumber (Cs), and watermelon (Cl). We generated the phylogenetic tree by using MEGA 7 software with the neighbor-joining method and bootstrap values are shown at each node. Accession numbers for *Arabidopsis thaliana* and *Oryza sativa* are taken from Ying *et al*. [25], and those for *Cucumis sativus* are as follows: *CsTRX h1* (CsaV3_6G046770), *CsTRX h2* (CsaV3_3G041740), *CsTRX h3* (CsaV3_2G029110), *CsTRX h4/1* (CsaV3_3G045510), *CsTRX h4/2* (CsaV3_5G008650), *CsTRX h4/3* (CsaV3_5G008560), *CsTRX h4/4* (CsaV3_5G008640), *CsTRX h5/1* (CsaV3_2G029120), *CsTRX h5/2* (CsaV3_2G029130), *CsTRX h7* (CsaV3_5G032200), *CsTRX h9* (CsaV3_4G027270), *CsTRX h10* (CsaV3_5G039280), and *CsCXXS1* (CsaV3_1G011310). (B) Multiple sequence alignment of h-type ClTRX proteins identified in *C. lanatus* using Geneious software. The sequences highlighted by the red box indicate the conserved WC(G/P)PC domain.

The h-type TRXs include the largest family of cytosolic TRXs and exhibit diverse functions [24]. We constructed a phylogenetic tree of the h-type TRXs from *Cucumis sativus* (Cs) and *C. lanatus* (Cl) with the well-characterized h-type TRXs from *Arabidopsis* (At) and *Oryza sativa* (Os) [25]. The h-type TRXs were divided into five subgroups as shown in Fig. 1A. Multiple sequence alignment analysis revealed that the h-type ClTRXs shared the conserved catalytic site, WCXXC, with the exception of the two proteins, ClCXXS1/1 and ClCXXS1/2, that are the orthologs of AtCXXS1 (Fig. 1B).

### A cold-induced ClTRX h2 localized at the plasma membrane is responsible for chilling tolerance in watermelon

**Figure 2:**
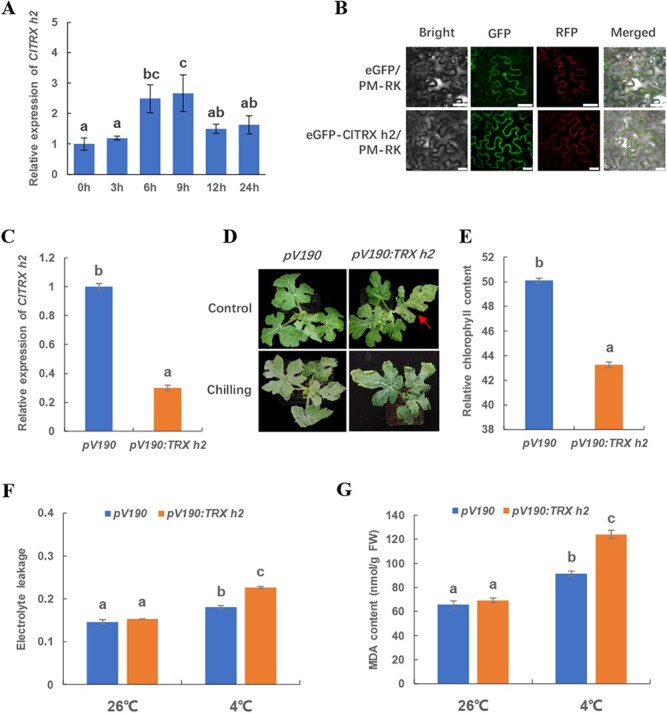
Function of ClTRX h2 responding to chilling in watermelon. (A) Time course of expression of *ClTRX h2* upon chilling stress in watermelon plants. (B) Subcellular localization of ClTRX h2. The GFP signal was merged with that of the plasma membrane marker PM-RK under a confocal microscope (merged). Scale bars, 30 μm. (C) Gene silencing efficiency of *ClTRX h2* in VIGS plants. (D) Phenotypes of *ClTRX h2*-silenced plants under normal (control) and chilling conditions. The arrow indicates a leaf with chlorosis symptoms. (E) Relative chlorophyll content (SPAD index) in *ClTRX h2*-silenced plants grown under normal conditions. (F) Changes in electrolyte leakage upon *ClTRX h2* silencing with and without chilling stress. (G) Changes in MDA content upon *ClTRX h2* silencing with and without chilling stress. Samples for phenotype analysis, electrolyte leakage, and MDA content were taken at 48 h after chilling treatment. Values are means of four biological replicates (± standard errors). Different letters represent significant differences at *P* < 0.05.

AtTRX h2, a subgroup II h-type TRX, was previously reported to affect cold tolerance in *Arabidopsis* [26]. ClTRX h2 (Cla017030) is the homolog of AtTRX h2 in watermelon (Fig. 1A). We found that *ClTRX h2* was dynamically induced when responding to chilling stress. *ClTRX h2* was significantly upregulated after chilling treatment for 6 hours, and the highest level of its transcripts was detected after 9 hours (Fig. 2A). To elucidate the location of ClTRX h2, we transiently expressed ClTRX h2 fused with enhanced GFP (eGFP) directed by the *CaMV35S* promoter in tobacco epidermal cells. The GFP fluorescence signal of the eGFP–ClTRX h2 fusion protein was merged with the plasma-membrane-localized red fluorescence reporter protein, confirming that the subcellular localization of ClTRX h2 was at the plasma membrane (Fig. 2B).

To fully investigate ClTRX h2 responding to chilling stress, we used virus-induced gene silencing (VIGS) to obtain *ClTRX h2*-silenced plants in watermelon. The expression of *ClTRX h2* in the *pV190:TRX h2* plants was reduced by 70% in comparison with the *pV190* control plants, suggesting that VIGS of *ClTRX h2* functions well in watermelon (Fig. 2C). The silencing of *ClTRX h2* resulted in mild leaf chlorosis as evidenced by decreased relative chlorophyll content (SPAD index) in the *pV190:TRX h2* plants compared with the control (Fig. 2D and E). To examine the phenotype of ClTRX h2 in the chilling stress response, the *pV190* control and *ClTRX h2*-silenced plants were placed at 4°C for 48 hours. *ClTRX h2* silencing caused more leaf injury than was seen in the *pV190* plants after chilling treatment (Fig. 2D). Additionally, the electrolyte leakage and MDA content were increased by 23.3% and 47.6% in the *pV190* control plants after chilling stress, while they were increased by 39.3% and 79.7% in the *ClTRX h2-*silenced plants under the same chilling treatment, respectively (Fig. 2F and G). In summary, these results suggested that ClTRX h2 positively regulates the chilling tolerance of watermelon plants.

### ClTRX h2 inhibits chilling-induced activation of ClMPK3

**Figure 3:**
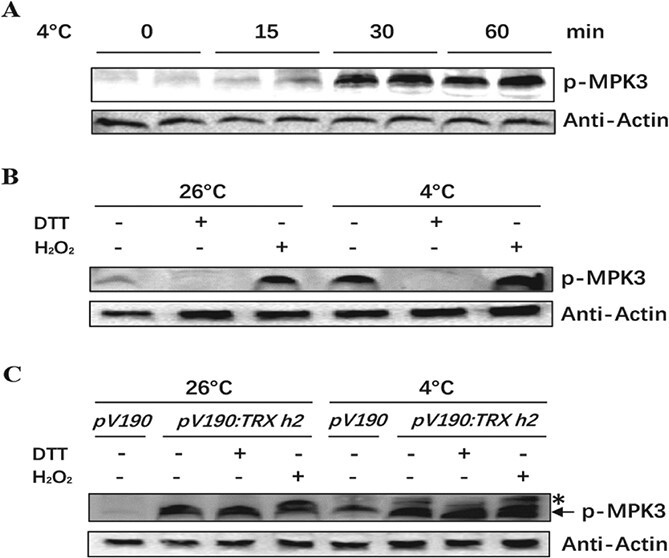
ClTRX h2 inhibits chilling-activated ClMPK3 phosphorylation in watermelon plants. (A) Chilling actives the phosphorylation of ClMPK3. Watermelon plants with six leaves were challenged with 4°C for 0, 15, 30, and 60 minutes. (B) The reductant DTT inhibited but oxygenant H_2_O_2_ promoted ClMPK3 phosphorylation. Watermelon plants with six leaves were treated under 4°C for 60 minutes. DTT and H_2_O_2_ were applied to detect the changes in ClMPK3 phosphorylation. (C) Effects of DTT and H_2_O_2_ on ClMPK3 phosphorylation in *ClTRX h2*-silenced plants upon chilling stress. Total proteins of each sample were normalized and immunoblotting assays were performed using a phospho-p44/42 MAPK antibody. The upper band indicated by the asterisk in (C) represents non-specific recognition of ClMPK3 against the phospho-p44/42 MAPK antibody. Actin was applied as a control.

To determine whether MAPK is phosphorylated in response to chilling stress, wild-type watermelon seedlings with six leaves were exposed to 4°C for 0, 15, 30, or 60 minutes. Immunoblotting was conducted to detect the phosphorylated/activated form of MAPK. Western blot results showed that ClMPK3 (the band was confirmed by mass spectrum detection) was significantly activated after chilling treatment for 30 minutes and lasted for 60 minutes (Fig. 3A; Supplementary Data Fig. S1). We also assessed the activation of ClMPK3 in the presence of dithiothreitol (DTT, as a reductant) or H_2_O_2_ (as an oxygenant) after exposure to 26°C and 4°C for 1 h, respectively. The phosphorylation of ClMPK3 completely disappeared in the presence of DTT, whereas H_2_O_2_ treatment induced ClMPK3 activation at 26°C in wild-type watermelon seedlings. Chilling activated ClMPK3 in the absence of DTT and H_2_O_2_, whereas exposure to H_2_O_2_ induced a more significant activation of ClMPK3 at 4°C. Again, the activation of ClMPK3 under chilling stress was compromised by DTT treatment (Fig. 3B). These results demonstrate a link between redox balance regulation and MAPK phosphorylation in response to chilling in watermelon plants.

To further clarify the function of ClTRX h2 in the activation of ClMPK3 in response to chilling stress, total protein was extracted from *pV190* and *pV190:TRX h2* plants exposed or not exposed to chilling followed by treatment with DTT or H_2_O_2_. We found that silencing of *ClTRX h2* increased the phosphorylation of ClMPK3 under 26°C conditions, but this increased phosphorylation was not obviously changed in the presence of DTT or H_2_O_2_ in the *pV190:TRX h2* plants (Fig. 3C). Furthermore, chilling activated ClMPK3 in the *pV190* control plants, whereas *ClTRX h2* silencing induced a more significant activation of ClMPK3 under chilling stress treatment (Fig. 3C). Similar to the normal condition treatment, ClMPK3 phosphorylation in the *pV190:TRX h2* plants after chilling stress showed no additive variations in the following treatment with DTT or H_2_O_2_ (Fig. 3C). These results indicate that ClTRX h2-mediated redox regulation is critical for the activation of ClMPK3 under chilling stress, and that both DTT-inhibited and H_2_O_2_-induced ClMPK3 activation are ClTRX h2*-*dependent in watermelon plants.

### ClTRX h2 interacts with ClMPKK5 and inhibits ClMPK3 activation *in vitro*

**Figure 4:**
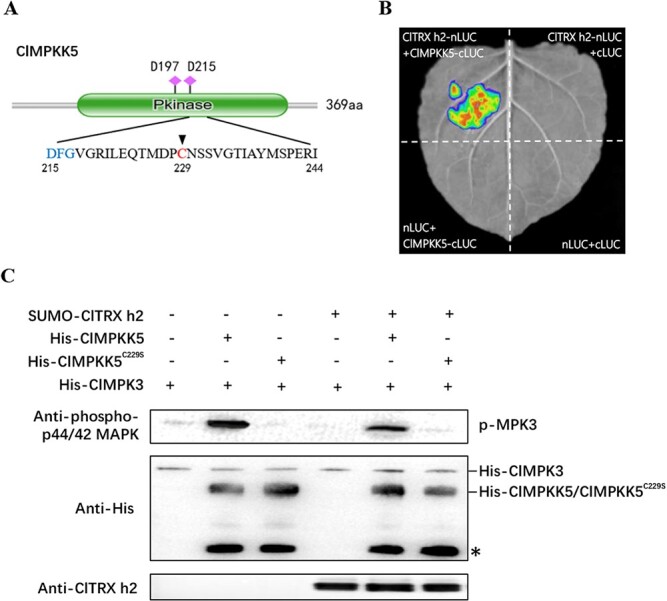
ClTRX h2 directly interacts with ClMPKK5 and inhibits ClMPK3 phosphorylation. (A) Diagram of cysteine (in red) and the magnesium-binding DFG motif (in blue) in ClMPKK5. (B) Interaction between ClTRX h2 and ClMPKK5 detected by LUC complementation. Tobacco leaves divided into four parts were separately infected with *Agrobacterium* strains containing the indicated constructs. The images were taken with a charge-coupled device (CCD) camera at 48 h post-inoculation. (C) ClMPK3 phosphorylation mediated by ClTRX h2 and ClMPKK5 *in vitro*. Phosphorylated ClMPK3 was detected using anti-phospho-p44/42 MAPK antibody (upper panel). Recombinant ClMPKK5/ClMPKK5^C229S^ and ClMPK3 proteins were detected by anti-His antibody (middle panel). The intense band indicated by the asterisk in the middle panel represents the degradation products of ClMPKK5/ClMPKK5^C229S^. Immunity to recombinant ClTRXh2 protein was produced by a synthesized anti-ClTRX h2 antibody (bottom panel).

Several studies have reported the importance of cysteine-dependent redox regulation of MAPK signaling cascades in the oxidative stress response of *Arabidopsis*, rice, and *Caenorhabditis elegans* [19, 27, 28]. Moreover, the cysteine residue is near the magnesium-binding DFG motif and located at the kinase activation domain, which is crucial for redox regulation and kinase activity [29]. Among the six ClMPKK proteins in watermelon, we found two cysteine residues near the DFG motif in ClMPKK3 (Cys-239) and ClMPKK5 (Cys-229), respectively (Supplementary Data Fig. S2A). To investigate whether ClTRX h2 regulates the MAPK signaling cascades, we then tested the interactions between ClTRX h2 and ClMPKK3/ClMPKK5 proteins by a yeast two-hybrid (Y2H) assay. The Y2H results showed that ClTRX h2 physically interacted with ClMPKK3 and ClMPKK5 (Supplementary Data Fig. S2B). Considering that MPKK4/MPKK5 was extensively reported to be responsible for the activation of MPK3 in *Arabidopsis* [22, 23, 30], we focused on ClMPKK5 as a potential target of ClTRX h2 (Fig. 4A). In addition to the interaction in the Y2H system, we also detected luciferase complementation (LUC) signals in tobacco leaves that co-infected with *Agrobacterium* strains that expressed ClTRX h2-nLUC and ClMPKK5-cLUC, but no signal was detected in the negative groups (ClTRX h2-nLUC and cLUC, nLUC and ClMPKK5-cLUC, nLUC and cLUC) (Fig. 4B). Thus, ClTRX h2 directly interacts with ClMPKK5.

To examine how ClTRX h2 regulates the MAPK signaling cascades, we purified recombinant SUMO-tagged ClTRX h2, His-tagged ClMPKK5/ClMPKK5^C229S^, and His-tagged ClMPK3, and carried out *in vitro* phosphorylation assays. Recombinant His-ClMPKK5 strongly activated ClMPK3 while mutation of the Cys-229 residue to Ser abrogated ClMPKK5 activation (Fig. 4C), suggesting that Cys-229 is essential for the ClMPKK5–ClMPK3 cascade. By contrast, we observed that recombinant SUMO-ClTRX h2 did not significantly change ClMPK3 phosphorylation in the absence of recombinant ClMPKK5, indicating that ClTRX h2 cannot directly regulate the phosphorylation of ClMPK3. Interestingly, recombinant His-ClMPKK5-activated ClMPK3 phosphorylation was substantially suppressed in the presence of SUMO-ClTRX h2 (Fig. 4C). Collectively, these results suggest that the ClMPKK5–ClMPK3 cascade in watermelon might be regulated by ClTRX h2 in a redox-dependent manner.

### ClMPK3 negatively regulates the chilling response by activating the CBF-dependent pathway

**Figure 5:**
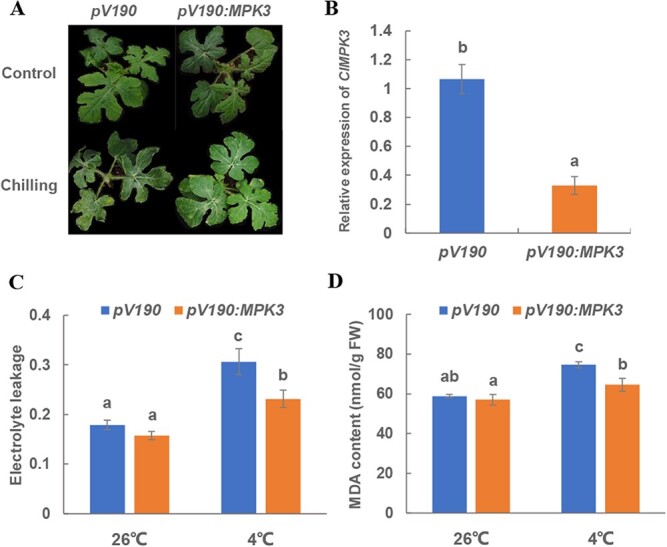
ClMPK3 negatively regulates chilling tolerance in watermelon. (A) Phenotypic analysis of *ClMPK3*-silenced plants in normal (control) and chilling conditions. (B) Gene silencing efficiency of *ClMPK3* in VIGS plants. (C) Electrolyte leakage and (D) content of MDA in *pV190* (control) and *ClMPK3*-silenced plants with and without chilling treatment. Samples for phenotype analysis, electrolyte leakage, and MDA content were taken after 48 hours of chilling stress. Values are means of four biological replicates (± standard errors). Different letters represent significant differences at *P* < 0.05.

To determine the function of ClMPK3 in response to chilling in watermelon plants, *pV190* control and *ClMPK3*-silenced plants were simultaneously treated with 4°C for 48 hours. As shown in Fig. 5A and B, *ClMPK3* silencing did not significantly change plant growth compared with *pV190* plants at the normal temperature (control). Chilling induced oxidative damage to the *pV190* leaves as indexed by 71.2% and 26.6% increases in electrolyte leakage and MDA content, respectively (Fig. 5A, C, and D). By contrast, the increases in electrolyte leakage and content of MDA were compromised in *ClMPK3*-silenced plants after chilling treatment (Fig. 5C and D), suggesting that ClMPK3 negatively regulates chilling tolerance in watermelon plants.

**Figure 6:**
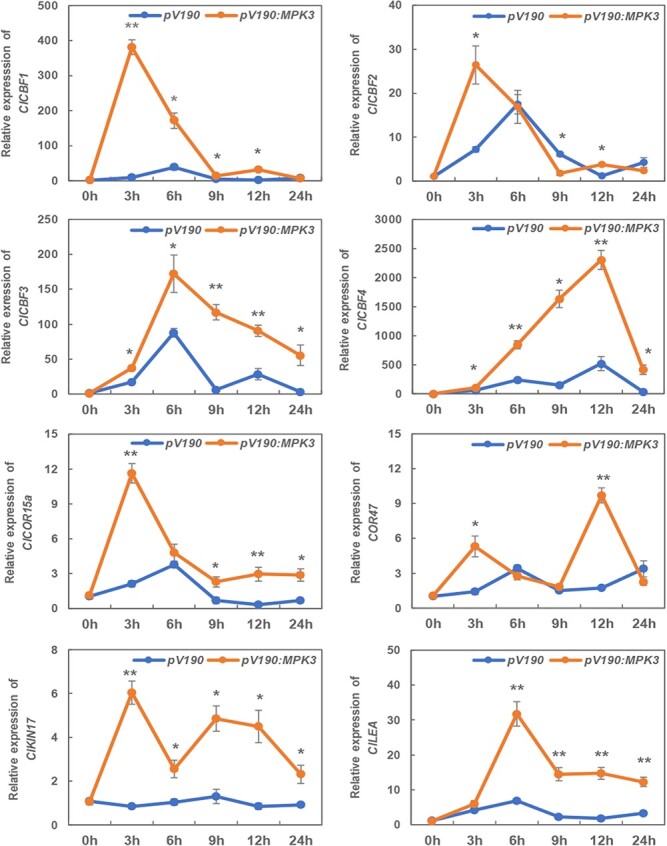
Silencing of *ClMPK3* induces the expression of *C-repeat/DREB binding factor* (*ClCBF*) and *cold-responsive* (*ClCOR*) genes differentially upon chilling stress. Leaf samples were taken at 0, 3, 6, 9, 12, and 24 hours following chilling treatment. Data are shown as the means of four biological replicates (± standard errors). ^*^ and ^**^ denote significant difference at *P* < .05 and *P* < .01, respectively, between the *pV190* control and *ClMPK3*-silenced plants.

The phosphorylation of MAPK is essential to the expression of *CBF*s [22, 23]. To evaluate whether ClMPK3-mediated chilling response is CBF-dependent, we analyzed the expression of *ClCBF*s and *ClCORs* in *pV190* and *pV190:MPK3* plants after 0, 3, 6, 9, 12, and 24 hours of chilling stress treatment (Fig. 6). The expression of *ClCBF1*/*2*/*3* and their corresponding targets, namely, *ClCOR15a*, *ClCOR47*, and *ClLEA*, was significantly increased after 6 hours of chilling treatment in the *pV190* control plants. For *ClCBF4*, the highest transcript level was observed after 12 hours of treatment in *pV190* control plants. For the *ClKIN17* gene, its transcripts showed relatively constant levels within 24 hours of chilling treatment in the *pV190* control plants. By contrast, *ClMPK3* silencing induced an earlier response of *ClCBF1* and *ClCBF2* when compared with *pV190* control plants, and their target genes, *ClCOR15a*, *ClCOR47*, and *ClKIN17*, were largely induced by chilling stress after 3 hours of treatment. In addition, the relative expression of *ClCBF3*, *ClCBF4*, and *ClLEA* was constantly higher in the *pV190:MPK3* plants in contrast to the *pV190* control plants from 6 h of chilling treatment. These results suggested that ClMPK3 negatively regulates chilling-responding gene expression mainly in a CBF-dependent manner in watermelon plants.

## Discussion

### ClTRX h2 positively regulates the response to chilling stress in watermelon plants

Plant cells have evolved a coordinated system to deal with high production rates of ROS and have formed a complex scavenging system to keep the redox status of the cell environment. The scavenging system mainly contains non-enzymatic compounds, such as ascorbic acid, glutathione (GSH), and various antioxidant enzymes [31]. Plant TRXs function in the abolishment of oxidative damage by providing reducing power to facilitate the reductases that detoxify lipid hydroperoxides or repair oxidized proteins [32]. A recent genome-wide transcriptional analysis of the typical and atypical TRXs involved in cold, osmotic, and drought stress in *Arabidopsis* reveal differential expression patterns of the 41 *TRX* genes in the shoots and roots following stress treatment at different times [9]. In this study, plasma membrane-localized ClTRX h2 was identified in watermelon (Fig. 2B). Unlike the published results showing that there were no variations in the mRNA and protein levels of the homologous AtTRX h2 in *Arabidopsis* between warm and cold conditions [18], we found that *ClTRX h2* was sensitive to chilling stress at the transcriptional level (Fig. 2A), indicating that *TRX*s responding to cold temperatures are plant species-dependent.

The labeling of target sulfhydryl groups or affinity chromatography using dysfunction of TRX proteins to screen targets facilitates the identification of TRX-dependent reductases and TRX-regulated enzymes that are associated with oxidative stress response [33–35]. For example, y-type TRXs in plastids regulate the redox status of monodehydroascorbate reductase, serving as major antioxidants to increase the tolerance to drought stress in plants [36]. Ectopic expression of the chloroplastic drought-induced *GhTRX134* from cotton (*Gossypium hirsutum*) increased drought tolerance in *Arabidopsis* by promoting the activities of catalase, superoxide dismutase, and peroxidase [37]. Overexpression of tomato *SlTrxh* in tobacco increased tolerance of excess nitrate stress through an interaction with the SlPrx protein [38]. Consistent with these studies, our results support the participation of ClTRX h2 in the antioxidant defense process under chilling conditions (Fig. 2D, F, and G). We thus consider that ClTRX h2 is a key factor in protecting watermelon plants from oxidative damage during chilling stress.

### Chilling-induced phosphorylation of ClMPK3 is correlated with ClTRX h2 function

Post-translational modifications could influence the redox state of cysteine residues, which can promptly and reversibly regulate protein function to manipulate biological processes. In *C. elegans*, the well-known p38 MAPK signaling cascade is composed of NSY-1 (ASK1), SEK-1 (MKK3/MKK6), and PMK-1 (p38 MAPK) [39]. The conservative cysteine-to-serine mutation at either the C213 of SEK-1 or the C173 of PMK-1 leads to the inhibition of ROS-induced PMK-1 activation [36], indicating the unique importance of cysteine in the MAPK signaling cascade. Similarly, the functional role of redox-sensitive cysteine residues was confirmed in rice by site-directed mutagenesis of OsMPK3 or OsMPK6 [40]. In our study, chilling-induced ClMPK3 phosphorylation was abolished in DTT-incubated protein extracts (Fig. 3A and B), indicating that redox-dependent MAPK phosphorylation occurred during chilling stress in watermelon plants. Interestingly, we further observed protein–protein interaction between ClTRX h2 and ClMPKK5, and the ClMPKK5 contains a cysteine residue close to the DFG motif, which is important for kinase activity (Fig. 4A and B). Our results show that the ClMPK3 activation by ClMPKK5 is abolished by a point mutation of the Cys-229 residue to Ser in ClMPKK5 (Fig. 4C), consistent with the previous finding that two cysteines (Cys-246 and Cys-266) located near the DFG motif are critical determinants for MKK4 activation in endothelial cells [41]. Thus, the present study suggests that chilling-induced MAPK signaling may be mediated, in part, through the redox regulation of ClTRX h2 by interacting with ClMPKK5. In animals, Trxs regulate the redox balance of multiple transcription factors [42]; Trx protein is an inhibitor of ASK1/MAPKKK5, which functions upstream of JNK and p38 MAPK, and is involved in the apoptosis process [43]. Trx binds directly to ASK1, depending on the redox status of Trx. Our results indicate increased activation of ClMPK3 in *ClTRX h2*-silenced plants under normal or chilling conditions in the absence or presence of DTT and H_2_O_2_ (Fig. 3C). This suggests that ClMPK3 phosphorylation under chilling treatment is specifically related to the function of ClTRX h2 in watermelon. Chilling-induced oxidation of ClTRX h2 by ROS likely releases ClMPKK5, which subsequently phosphorylates ClMPK3 to further regulate the chilling response in watermelon plants.

### Interaction of ClTRX h2 with MAPK cascades likely mediates chilling-induced *ClCBF* expression

By genetic and molecular analyses, CBFs are identified as pivotal transcription factors that function in cold signal transduction to trigger downstream gene expression [21]. CBFs have been characterized from various crops, including rice, wheat, barley, maize, and tomato [44, 45]. In our previous study we identified four CBFs in watermelon [46]. *CBF* expression is sensitive to cold stress, and overexpression of *CBF* genes triggers the expression of *cold-responsive* (*COR*) genes and finally increased cold tolerance in many plant species [47–49]. For example, *CBF*-like transcripts and *COR* genes accumulate quickly (within 15–30 minutes) and differentially in response to cold stress in *Arabidopsis*, *Brassica napus*, rye, wheat, and tomato [50]. Here, we discovered that *ClCBF1*/*2*/*3* was consistently induced after 6 hours of chilling stress in *pV190* control plants, whereas the highest expression of *ClCBF4* was seen after 12 hours of chilling stress treatment (Fig. 6), supporting the notion of a positive role of *CBF1*/*2*/*3* in the response to chilling stress in watermelon plants, similar to *Arabidopsis* [51, 52].

**Figure 7:**
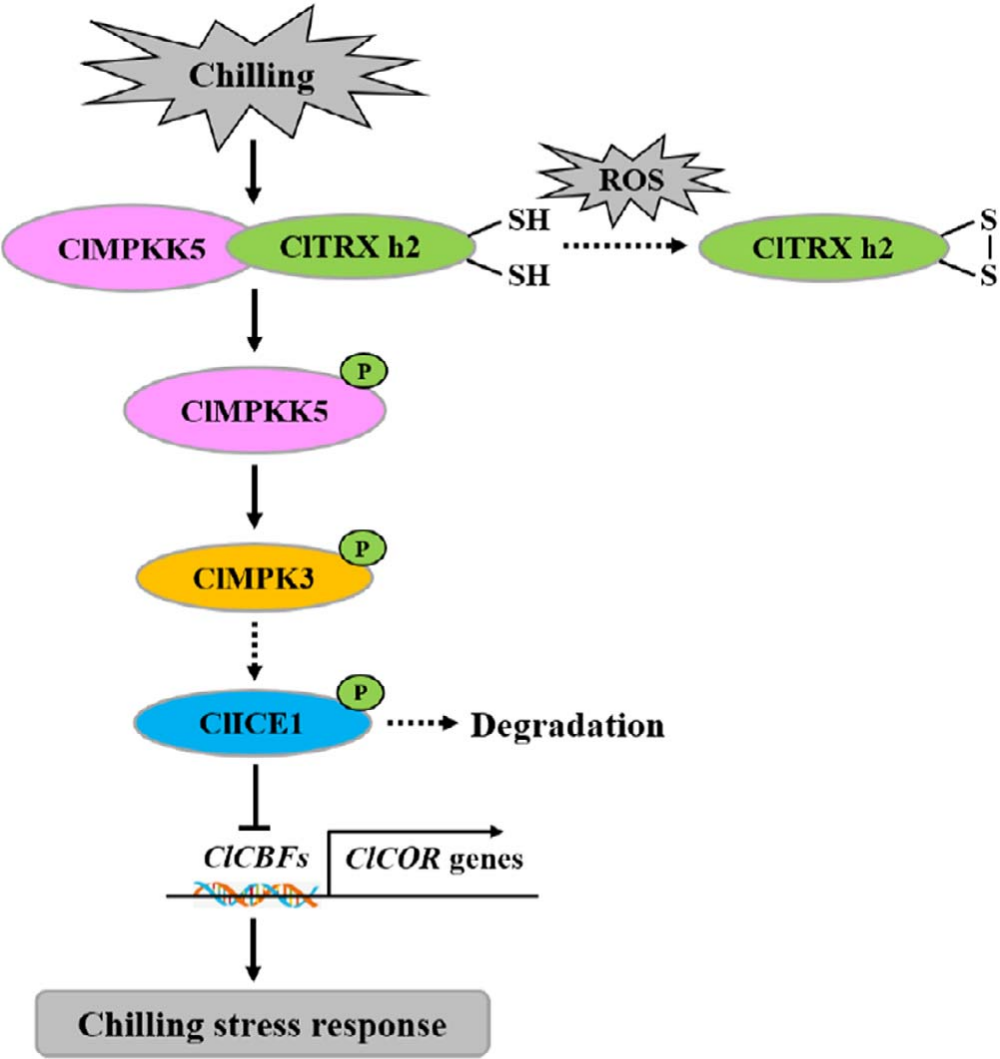
A working model of the chilling-induced ClTRX h2–ClMPKK5–ClMPK3–ClCBF module in watermelon plants. ROS induced by chilling stress are likely to accelerate the oxidation and dissociation of ClTRX h2 from ClMPKK5 protein, thereby leading to enhanced phosphorylation of ClMPK3 that is catalyzed by ClMPKK5. ClICE1 may serve as an endogenous phosphorylation target of ClMPK3, which fails to initiate the activation of *ClCBF* and *ClCOR* genes due to phosphorylation-dependent degradation of ClICE1 in the response to chilling stress.

ICE1 is an important transcriptional activator of *CBF* expression, belonging to the MYC-like basic helix–loop–helix (bHLH) transcription factor family [53]. In plants, ICE1 could directly interact with MYC-binding sites (CANNTG) in the promoters of *CBF1*/2/*3* to induce their expression when under cold stress challenge [54]. Recently, several protein kinases, including MAPKs, were reported to regulate the CBF signaling pathway during plant cold acclimation. In *Arabidopsis*, MPK3 and 6 are negative regulators in the cold response, and MPK3 and 6 phosphorylate ICE1 at the beginning of cold signaling, leading to polyubiquitination and degradation of ICE1 [22, 23]. Similarly, our results showed increased expression of *ClCBF*s and their target genes (i.e. *ClCOR15a*, *ClCOR47*, *ClKIN17*, and *ClLEA*) in *ClMPK3*-silenced plants compared with *pV190* control plants under chilling stress (Fig. 6), demonstrating the negative role of ClMPK3 in the chilling tolerance of watermelon plants (Fig. 5) [22, 23]. On the contrary, chilling-induced activation of *ClCBF* and *ClCOR* genes at an early stage (3 hours after chilling stress) was compromised in *ClTRX h2*-silenced plants compared with the *pV190* control (Supplementary Data Fig. S3), further demonstrating the role of ClTRX h2 in regulating the CBF–COR pathway under chilling conditions. It should be noted that a previous study evidenced that OsMPK3 phosphorylates and stabilizes OsICE1, and positively regulates chilling tolerance in rice [55]. Interestingly, our study indicates that ClMPK3 is a negative regulator of the response to chilling in watermelon plants. These findings demonstrate that MPK3 plausibly has distinct or even opposite roles in the regulation of cold signaling in different plant species. Overall, our results suggest that watermelon ClTRX h2 inhibits ClMPK3 phosphorylation in a redox-dependent manner by interacting with ClMPKK5, which synergistically regulates *ClCBF* and *ClCOR* gene expression under chilling stress conditions (Fig. 7). Our study updates the function of TRX in chilling stress tolerance of watermelon plants, and enriches the regulation layer of the cold-responsive signaling pathway in which TRX manipulates the cellular redox status to trigger MAPK cascade signaling.

## Materials and methods

### Identification of the ClTRX family members

The thioredoxin domain model (PF00085) was downloaded from the Pfam database (http://pfam.xfam.org). HMM tools (http://hmmer.janelia.org/) were used to identify TRX candidates contained in the genomes of watermelon (97103) v1 and cucumber (‘Chinese Long’) v3 (Cucurbit Genomics Database, CuGenDB). The prediction of protein subcellular locations was performed at SUBA4 (https://suba.live/). We used the Expasy Server with a compute tool to calculate the isoelectric point (pI) and protein molecular weight (Mw) at https://web.expasy.org/compute_pi/. A phylogenetic tree was generated with the distance-based neighbor-joining method in MEGA 7 software. Multiple sequence alignment was done using Geneious software.

### Virus-induced gene silencing

Watermelon seeds (‘Zhengkang 2’) were germinated optimally in the dark, and sown in a mixed medium containing peat and vermiculite (2:1, v/v) under the photosynthetic photon flux density (PPFD) of 300 μmol m^−2^ s^−1^ (12-hour photoperiod). The temperature condition was kept at 28°C/18°C (light/dark).

To silence the *ClTRX h2* and *ClMPK3* genes, fragments of 148 and 246 bp of the corresponding cDNA were introduced into the BamHI cloning site of the pV190 vector to generate *pV190:TRX h2* and *pV190:MPK3*, respectively. The PCR amplification primers were designed from distinguishable sequences as shown in Supplementary Data Table S1. The *pV190:TRX h2* or *pV190:MPK3* construct was validated by sequencing and finally transfected into the *Agrobacterium tumefaciens* strain GV3101. *Agrobacterium* infection was carried out using watermelon seedlings at the 10-day stage as described previously [56]. An *Agrobacterium* culture expressing pV190 vector was used as a control. Three weeks later, quantitative real-time PCR (qPCR) was conducted to test the efficiency of the targeted gene-silencing before the gene-silenced plants were applied in the subsequent experiments.

### Relative chlorophyll content

We used a Chlorophyll Meter (SPAD-502, Minolta Camera Co., Ltd, Osaka, Japan) to measure the relative chlorophyll content (SPAD index) of the fifth leaf of each plant.

### Chilling stress treatment

Well-grown watermelon seedlings at the stage of six leaves were randomly divided into two groups and placed into growth chambers at 26 and 4°C with constant light of 300 μmol m^−2^ s^−1^ (Saifu DGX-260, Ningbo, China), respectively. Next, the leaf samples were collected at the indicated times after chilling stress treatment to test electrolyte leakage, malondialdehyde (MDA) content, protein phosphorylation, and gene expression.

### Evaluation of electrolyte leakage and malondialdehyde content

Electrolyte leakage was detected as described previously [46]. MDA content was quantified to show membrane lipid peroxidation following the reported method [47] after minor modifications. In brief, 0.3-g leaf samples were ground into powder and dissolved in 5 ml buffer containing 10% trichloroacetic acid (TCA). The fully mixed homogenates were centrifuged at 12 000 *g* (4°C) for 20 minutes, and the supernatants were transferred and reserved for MDA measurement. The samples were mixed with a reaction buffer containing TCA (10%) and 2-thiobarbituric acid (TBA, 0.65%) and incubated at 95°C for 25 minutes. MDA content was determined by measuring absorbance at 450, 532, and 600 nm.

### Detection of subcellular localization of ClTRX h2

The coding sequence (CDS, 426 bp) of *ClTRX h2* (Cla017030) without the stop codon was amplified by PCR using 2 × Super Pfx MasterMix (CW Biotech, Inc., Beijing, China). The amplified products were introduced into the StuI site of the pH7LIC5.0-N-eGFP vector with a commercial One Step Cloning Kit (Vazyme, Nanjing, China) to produce the eGFP–ClTRX h2 fusion protein expression construct. The reconstructed vector or empty pH7LIC5.0-N-eGFP was transformed into *A. tumefaciens* strain GV3101, and then infiltrated into tobacco leaves as described in a previous study [58]. The GFP fluorescence signal was monitored using a Leica SP8 confocal microscope (Leica, Wetzlar, Hessen, Germany) with the red fluorescence localization reporter vector (PM-RK) as the plasma membrane marker.

### Yeast two-hybrid analysis

For the protein–protein interaction, the CDSs of *ClTRX h2* and *ClMPKK3/ClMPKK5* were cloned into the SfiI site of a bait vector, pBT3-STE (pBT3-STE-ClTRX h2), and a prey vector, pPR3-N (pPR3-N-ClMPKK3/ClMPKK5), respectively. The constructed bait and prey vectors were co-transformed into the yeast strain NMY51. The yeast cells were cultured overnight at 30°C for 2 days on SD/−Leu/−Trp (SD/−LT) and SD/−Leu/−Trp/−Ade/-His (SD/–LTAH) medium, respectively. The concentration of the yeast colonies was adjusted to an OD_600_ of 0.6. To determine the mutual interaction between the two targeted proteins, we transferred the yeast cells to SD/–LTAH solid medium containing 1 mM 3-AT and diluted 10^−1^, 10^−2^, and 10^−3^ to produce a concentration gradient. The groups of pBT3-STE-ClTRX h2 and pPR3-N as well as pBT3-STE-ClTRX h2 and pOST1-NubI were set as negative and positive controls, respectively.

### Luciferase complementation assay

The CDSs of *ClTRX h2* and *ClMPKK5* were separately cloned into pCAMBIA-nLUC and pCAMBIA-cLUC to obtain the fusion constructs pCAMBIA-ClTRX h2-nLUC and pCAMBIA-ClMPKK5-cLUC [59]. *A. tumefaciens* GV3101 carrying nLUC and cLUC empty vector or recombinant plasmids were co-transfected into tobacco leaves after equal mixing. After 48 hours of infiltration, the leaves were treated with luciferin for firefly luciferase (LUC) signal detection using a charge-coupled device (CCD) imaging system (NightSHADE evo LB 985 N, Berthold, Germany).

### Protein extraction and phosphorylation detection

Leaf samples (0.3 g) were sampled and ground into powder in liquid nitrogen to extract protein. The total proteins were extracted with a buffer containing 125 mM Tris–HCl (pH 8.8), 1% SDS (w/v), 10% glycerol (v/v), 50 mM Na_2_S_2_O_5_, 5 mM EDTA, 10 mM Na_3_VO_4_, 1% Triton X-100, 50 mM β-glycerophosphate, and phosphatase inhibitor cocktail set II. The homogenates were mixed and centrifuged at 13 000 *g* for 10 minutes, and the supernatant was collected in a fresh tube. The protein concentration of each sample was measured using a protein assay kit (Beyotime Biotech, Inc., Shanghai, China), and normalized before protein gel electrophoresis. Denatured protein extracts were loaded and separated by 12% SDS–PAGE, and actin was used as a control. Protein phosphorylation was detected using a phospho-p44/42 MAPK (Erk1/2) (Thr202/Tyr204) antibody (Cell Signaling Technology, Danvers, MA, USA). Horseradish peroxidase-linked secondary antibody (Cell Signaling Technology, Danvers, MA, USA) was used to recognize the antigen, and finally the antigen–antibody complexes were tested using an enhanced chemiluminescence kit (Thermo, Waltham, MA, USA) following the manual. The phosphorylated proteins were identified by mass spectrometry (Wuhan Yunzhike Biotechnology Co., Ltd, Wuhan, China).

### *In vitro* protein kinase assay

To obtain recombinant protein *in vitro*, the CDS of ClTRX h2 removing the stop codon was cloned into the pET28b vector consisting of a SUMO tag. ClMPKK5, ClMPKK5^C229S^, or ClMPK3 was cloned into pET28b containing a His tag. All constructs were transformed into *Escherichia coli* strain BL21. Each transformant was cultured at 37°C in LB medium containing 50 μg/ml kanamycin until OD_600_ reached 0.4–0.6, and isopropyl-β-d-thiogalactopyranoside (IPTG) was subsequently added to a final concentration of 1 mM. *E. coli* cells with pET28b-ClMKK5/ClMKK5^C229S^ were induced at 37°C for 4 hours, while those with pET28b-ClMPK3 and pET28b-ClTRX h2 were induced at 16°C for 16 hours. The SUMO- and His-tagged recombinant proteins were purified using Ni Sepharose™ High Performance (GE Healthcare, Uppsala, Sweden).

An *in vitro* kinase assay was carried out using previously reported methods [19, 41]. In brief, 2 μg of recombinant SUMO-ClTRX h2, 5 μg of recombinant His-ClMKK5/ClMKK5^C229S^, and 5 μg of recombinant His-ClMPK3 (substrate protein) were incubated in 50 μl of kinase reaction buffer (50 mM Tris–HCl, pH 7.5, 20 mM MgCl_2_, 0.1 mM Na_3_VO_4_, 1 mM EGTA) containing 20 μM ATP at 30°C for 30 minutes. The phosphorylation reaction was blocked by adding SDS–PAGE sample buffer. ClMPKK5, ClMPKK5^C229S^, and ClMPK3 proteins were detected by immunoblot analysis using anti-His antibody (ABclonal, AE086). ClTRX h2-specific antibody was produced against two synthetic peptides (HFNSAQESSKLM and KIQKHRSASG) corresponding to the N terminus and C terminus of ClTRX h2, respectively (AtaGenix Laboratories Co., Ltd, Wuhan, China). Phosphorylated ClMPK3 was detected with the anti-phospho-p44/42 MAPK (Erk1/2) (Thr202/Tyr204) antibody [23].

### Total RNA extraction and qPCR assays

The total RNAs were isolated from leaf tissues with TransZol reagent (TransGen Biotech Inc., Beijing, China) according to the protocol. The cDNA for qPCR was reverse-transcribed from 1 μg of total RNA using the HiFiScript gDNA Removal cDNA Synthesis Kit (CW Biotech, Inc., Beijing, China). The PCR reactions were run in a 10-μl reaction system containing 2 × TransStart™ TOP Green qPCR SuperMix (TransGen Biotech Inc., Beijing, China). The QuantStudio 7 Flex Real-time PCR System (Applied Biosystems, Foster City, CA, USA) was used to perform PCR amplification. *ClCAC* was used as an internal reference gene [60]. The specific primers for the targeted genes are presented in Supplementary Data Table S1. The relative expression of genes was calculated using the formula proposed by Livak and Schmittgen [61].

### Statistical analysis

The completely randomized block design with four biological replicates was adopted for the experiment. In each replicate there were 12 well-grown watermelon seedlings. We used the SAS statistical package to conduct the statistical analysis. Differences among the means of indicated treatments were determined using Tukey’s test and assigned a significance level of *P* < 0.05 or *P* < 0.01.

## Acknowledgements

This research was funded by the National Natural Science Foundation of China (32172551, 32002046), the Fundamental Research Funds for the Central Universities (2662020YLPY003), the China Agriculture Research System of MOF and MARA (CARS-25), and the Natural Science Foundation of Hubei Province (2019CFA017). Dr Qinsheng Gu from Zhengzhou Fruit Research Institute, Chinese Academy of Agricultural Sciences, provided the pV190 vector and watermelon seeds (Zhengkang 2).

## Author contributions

F.C. designed the research; A.Q.X., N.N.W., H.H., and S.Z. conducted the experiments; A.Q.X. and F.C. analyzed the data and wrote the manuscript; Y.H., Q.S.K., and Z.L.B. supervised the study; W.F.N provided intellectual support and all the authors revised the manuscript.

## Data availability

All the supplementary data relevant to this study are presented online.

## Conflict of interest

The authors declare that they have no conflicts of interest.

## Supplementary data

Supplementary data is available at *Horticulture Research* online.

## Supplementary Material

Web_Material_uhac256
