# Supplementary material for: Thioredoxin h2 inhibits the MPKK5-MPK3 cascade to regulate the CBF–COR signaling pathway in Citrullus lanatus suffering chilling stress
Source: Hortic Res. 2022 Nov 21;10(2):uhac256. doi: 10.1093/hr/uhac256 (PMC9907054; doi:10.1093/hr/uhac256)

*Supplementary Material*

Thioredoxin h2 inhibits MPKK5-MPK3 cascade to regulate the CBF-COR signaling pathway in *Citrullus lanatus* suffering chilling stress

Running title: Thiol-based redox regulation under chilling stress

Anqi Xu^1^, Nannan Wei^1^, Hao Hu^1^, Shu Zhou^1^, Yuan Huang^1^, Qiusheng Kong^1^, Zhilong Bie^1^, Wen-Feng Nie^2,*^, Fei Cheng^1,*^

^1^ *Key Laboratory of Horticultural Plant Biology, Ministry of Education, College of Horticulture and Forestry Sciences, Huazhong Agricultural University, Wuhan 430070, China*

^2^ *Department of Horticulture, College of Horticulture and Plant Protection, Yangzhou University, Yangzhou 225009, China*

^*^Corresponding author: Fei Cheng (feicheng@mail.hzau.edu.cn); Wen-Feng Nie (wfnie@yzu.edu.cn)

**Table S1. List of primer sequences used in this study.**

| Gene name | Forward primer 5’-3’ | Reverse primer 5’-3’ |
| --- | --- | --- |
| For VIGS constructs | | |
| *pV190:TRX h2* | acttaatggatcc  TTCTTCTATCAAGGATCGG | ctataactggatcc  GATTCCTGAGCGGAATTGAAG |
| *pV190:MPK3* acttaatggatcc ctataactggatcc  ACTCAGAGGACTCATCGCTG GCAACAACACCAGCCAACTG  For subcellular localization | | |
| pH7LIC5.0-N-eGFP-ClTRX h2 | attacgccgagg  TCATGGGAGTTCTTCTATCAAGGATCG | tagggagagg  TCAACCAGATGCTGATCGGT |
| For yeast two-hybrid assay | | |
| pBT3-STE-ClTRX h2 | attaacaaggccattacggcc  GGAGTTCTTCTATCAAGGATCGGA | aactgattggccgaggcggccc  ACCAGATGCTGATCGGTGC |
| pPR3-N-ClMPKK3  pPR3-N-ClMPKK5 | gtatcaacgcagagtggccattacggcc  ATGGCGGGTCTTGAGGAACT  gtatcaacgcagagtggccattacggcc  ATGAGACCGCTCCAGCCG | atcgaattctcgagaggccgaggcggcc CTATTGAATTATATAAAGCTCTTGTTTGGC  atcgaattctcgagaggccgaggcggcc  TCAGGAAAGGCTGGAAGGATG |
| For luciferase complementation assay  pCAMBIA-ClTRX h2-nLUC tcccggggcggtacc gctctgcaggtcgac  ATGGGAGTTCTTCTATCAAGGATCG ACCAGATGCTGATCGGT  pCAMBIA-ClMPKK5-cLUC tcccggggcggtacc gctctgcaggtcgac  ATGAGACCGCTCCAGCCG GGAAAGGCTGGAAGGATG  For *in vitro* kinase assay  SUMO-ClTRX h2 ctcacagagaacagattggtggatcc tcaaactgcggatggctccactcgagg  ATGGGAGTTCTTCTATCAAGGA TTAACCAGATGCTGATCGGT  His-ClMPKK5 ctcacagagaacagattggtggatcc tcaaactgcggatggctccactcgagg  ATGAGACCGCTCCAGCCG TTAGGAAAGGCTGGAAGGATG  His-ClMPKK5^C229S^ ctcacagagaacagattggtggatcc tcaaactgcggatggctccactcgagg  ATGAGACCGCTCCAGCCG TTAGGAAAGGCTGGAAGGATG  His-ClMPK3 tggtgccgcgcggcagccatatg caagcttgtcgacggagctcgaattc  GTAAAGTGCTACCCTACTGTAAGTGAG TTATGCAAATTCTGGATTGAGTGC  For qRT-PCR | | |
| *ClTRX h2* (Cla017030) | CCACTTCAATTCCGCTCAGG | GACGGCTCCATTAGCCTACA |
| *ClMPK3* (Cla008291)  *ClCBF1* (Cla017719) | CTCTAGTGAGCTCGCCCATG  ACGAGTTATGTTGTCATCGTT | GGCCCTCCAGTAACCTCAAC  CTCCTCCGACATCAAAGAA |
| *ClCBF2* (Cla011488) | AAGTGATTCTGGCGTCCAACCG | ATTCCTCCGGCGTACTCCTCTG |
| *ClCBF3* (Cla006212) | GAAGACTACTTCTCCAGTTCCG | CTGGCCGCCAACATAATATCC |
| *ClCBF4* (Cla002330) | TTCCTCCCCACTTTGCCAC | TTGCCGGAGTTCCGCTGTCGC |
| *ClCOR15a* (Cla013826)  *ClCOR47* (Cla014570) | TGGGATGGGTTGAAAGCAAG  ATGGCGCATTACCAATCTGG | TGTTCTCCGGTTGCATTTGC  TTCCATATTGGTCCGTCTCACG |
| *ClKIN17* (Cla004067)  *ClLEA* (Cla021202) | TGAGCAAGGATTTGGCTGAG  AACATGCCGAAGCCAGAG | TCAACACATGCTTGCCATCG  TGGAGACAGAGACATTGGC |
| *ClCAC* (Cla016178) | GAACTTGGCACCTGTCCTGT | GAACAGTGCAACAGCCTCAA |

**Table S2. Major information of ClTRXs in watermelon.**

| Gene name | Gene ID | Chr | Start | End | CDS | Amino acid | Subcellular localization | pI | Mw (kD) | Redox center |
| --- | --- | --- | --- | --- | --- | --- | --- | --- | --- | --- |
| ClTRX f | Cla002178 | Chr3 | 18076434 | 18078715 | 543 | 180 | Chloroplast | 8.97 | 19.31 | WCGPC |
| ClTRX m2 | Cla013870 | Chr8 | 15655705 | 15656346 | 549 | 182 | Chloroplast | 9.15 | 20.07 | WCGPC |
| ClTRX m3 | Cla016016 | Chr2 | 6288056 | 6290353 | 498 | 165 | Chloroplast | 6.72 | 18.60 | WCGPC |
| ClTRX m4 | Cla011786 | Chr7 | 10150667 | 10152834 | 534 | 177 | Chloroplast | 9.18 | 19.23 | WCGPC |
| ClTRX o | Cla009876 | Chr1 | 33702229 | 33705212 | 594 | 197 | Mitochondrion | 9.48 | 21.85 | WCGPC |
| ClTRX z | Cla022051 | Chr8 | 19892049 | 19894814 | 315 | 104 | Chloroplast | 4.22 | 12.00 | WCGPC |
| ClTRX h1 | Cla014255 | Chr1 | 29448490 | 29449557 | 367 | 121 | Cytosol | 5.55 | 13.37 | WCGPC |
| ClTRX h2 | Cla017030 | Chr10 | 20735643 | 20736775 | 429 | 142 | Cytosol, plasma membrane | 6.90 | 15.63 | WCGPC |
| ClTRX h3 | Cla022461 | Chr8 | 23841091 | 23842066 | 369 | 122 | Cytosol | 6.11 | 13.14 | WCGPC |
| ClTRX h4/1 | Cla020798 | Chr5 | 26677389 | 26677901 | 339 | 112 | Cytosol | 9.51 | 12.43 | WCGPC |
| ClTRX h4/2 | Cla003973 | Chr7 | 3095829 | 3096423 | 348 | 115 | Cytosol | 9.20 | 12.74 | WCGPC |
| ClTRX h4/3 | Cla003972 | Chr7 | 3081001 | 3082345 | 342 | 113 | Cytosol | 9.35 | 12.84 | WCGSC |
| ClTRX h5 | Cla022460 | Chr8 | 23836356 | 23837345 | 366 | 121 | Cytosol | 5.54 | 12.99 | WCPPC |
| ClTRX h7 | Cla010144 | Chr5 | 31731302 | 31731889 | 396 | 131 | Cytosol | 8.99 | 15.00 | WCGPC |
| ClTRX h9 | Cla012040 | Chr4 | 17540740 | 17543661 | 420 | 139 | Plasma membrane | 4.91 | 15.48 | WCGPC |
| ClTRX h10 | Cla016436 | Chr11 | 21305673 | 21307664 | 435 | 144 | Plasma membrane | 5.02 | 16.19 | WCRPC |
| ClCXXS1/1 | Cla001714 | Chr5 | 21711376 | 21712490 | 363 | 120 | Cytosol, plasma membrane, vacuole | 9.28 | 13.63 | WCTPS |
| ClCXXS1/2 | Cla019421 | Chr3 | 5051905 | 5053634 | 372 | 123 | Cytosol, plasma membrane, vacuole | 4.98 | 14.02 | WCMPS |

**Figure S1. Characterization of the ClMPK3 (Cla008291) protein using time-of-flight secondary ion mass spectrometry.** Two unique peptides were identified from the 72 kD immunized band using p44/42 MAPK (Erk1/2) (Thr202/Tyr204) antibody.


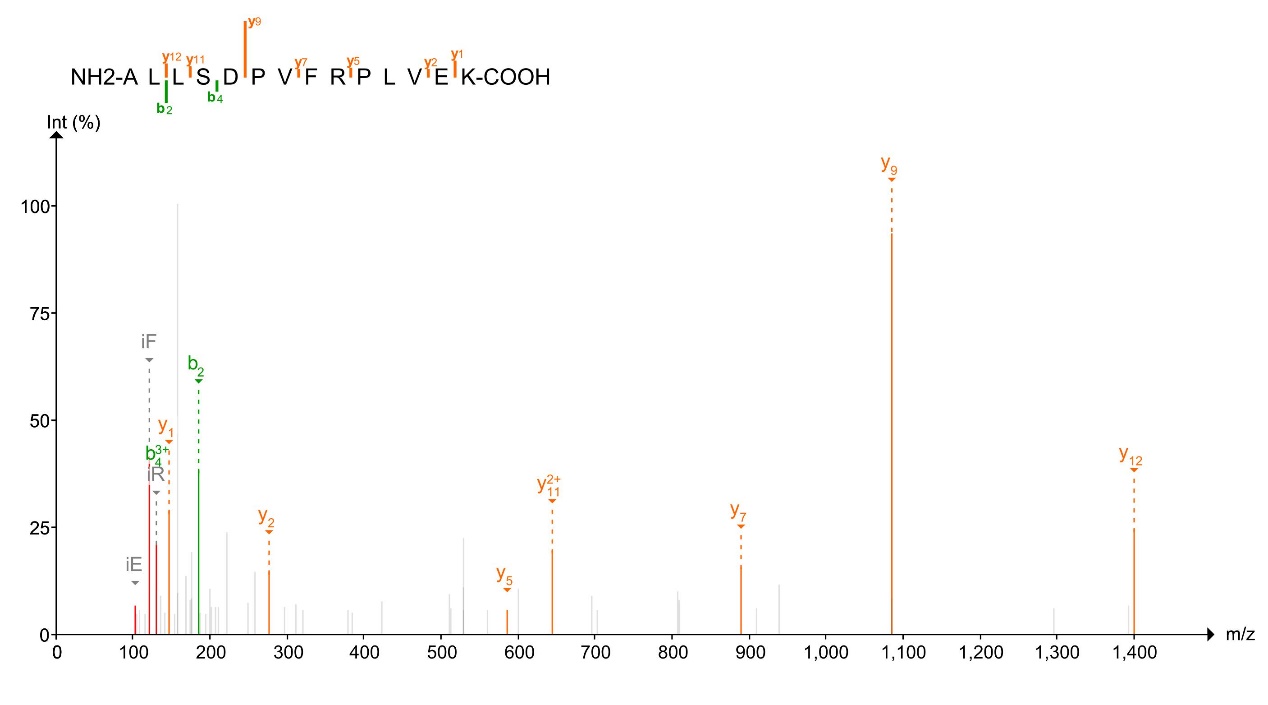


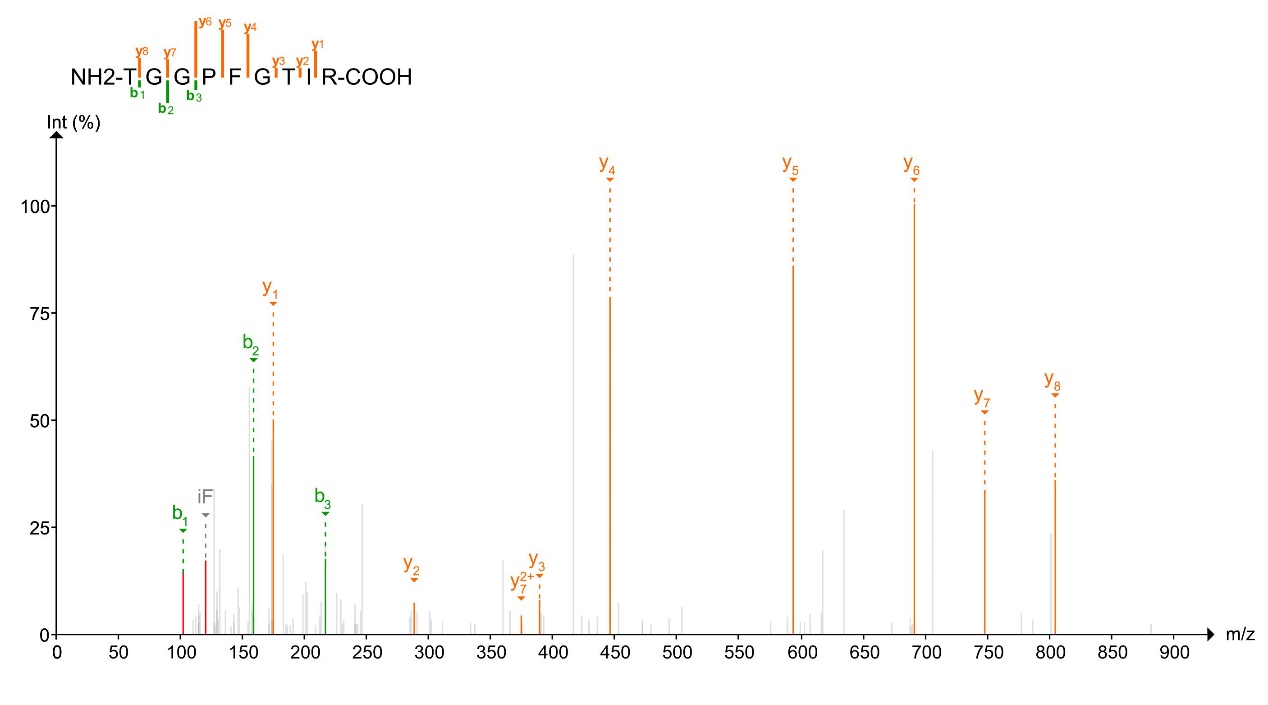


**Figure S2. ClTRX h2 directly interacts with ClMPKK3 and ClMPKK5. (A)** The diagram of cysteines (in blue) and the magnesium-binding DFG motifs (in black) in ClMPKK3 and ClMPKK5. **(B)** Interaction detection between ClTRX h2 and ClMPKK3/ClMPKK5 by Y2H assays. The two recombinant plasmids containing either pBT3-STE-ClTRX h2 and pPR3-N, or pBT3-STE-ClTRX h2 and pOST1-NubI were co-transformed into yeast strain NMY51 and set as negative and positive controls, respectively. Yeast cells were grown on SD/-Leu/-Trp (SD/-LT) and SD/-Leu/-Trp/-Ade/-His (SD/-LTAH) media.

**A**

*****


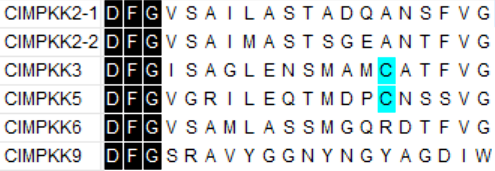


**B**

**Negative control**

**Positive control**

**ClMPKK3**

**ClMPKK5**

**SD/-LT**

**SD/-LTAH**

**10^0^ 10^-1^ 10^-2^ 10^-3^**

**10^0^ 10^-1^ 10^-2^ 10^-3^**


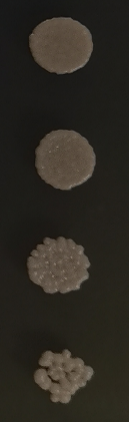

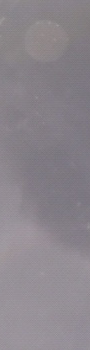

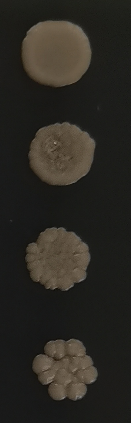

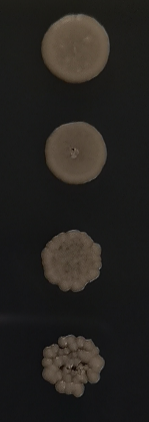

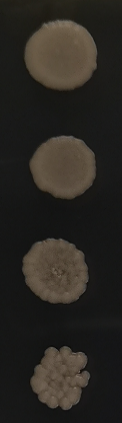

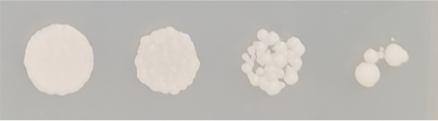

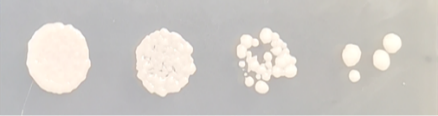

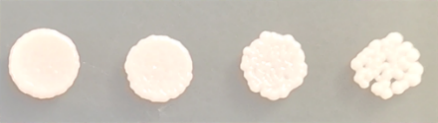


**Figure S3. Relative gene expression of *C-repeat/DREB binding factor* (*ClCBF*) and *cold-responsive* (*ClCOR*) genes in *pV190* control and *ClTRX h2*-silenced plants upon chilling stress.** Leaf samples were collected at 3 h after chilling stress. Data are shown as the means of four replicates with SEs. Different letters represent significant differences at *p* < 0.05.


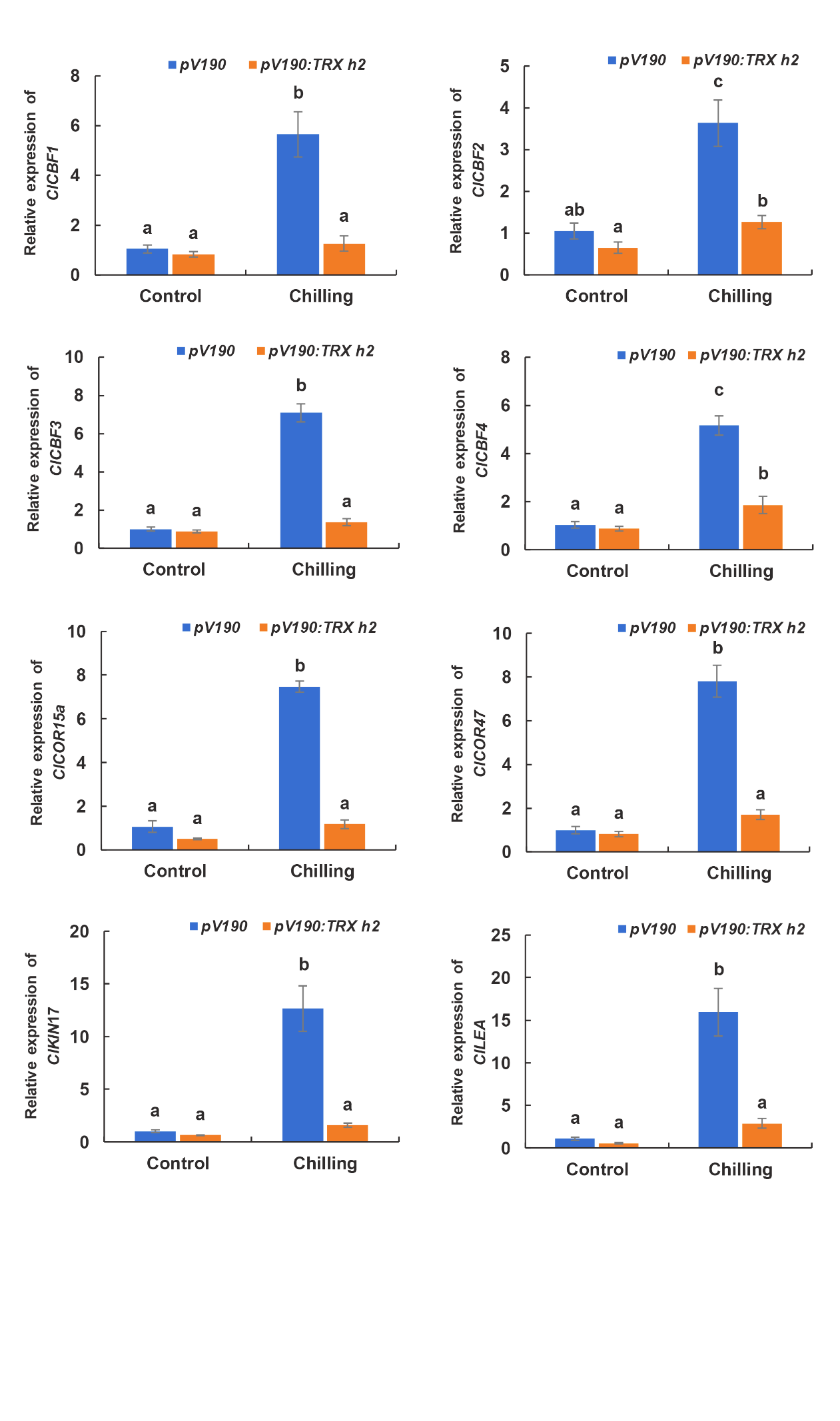

Supplement: Web_Material_uhac256 [file web_material_uhac256.zip › Supplementary material- 20221031.docx]
